# Evaluation of bone repair with platelet-rich fibrin following the extraction of impacted third molars - randomized clinical trial

**DOI:** 10.4317/medoral.25856

**Published:** 2023-06-18

**Authors:** Ewerton Daniel Rocha Rodrigues, Andrea dos Anjos Pontual, Rômulo Augusto de Paiva Macedo, Eduarda Nascimento, Belmiro Cavalcanti do Egito Vasconcelos

**Affiliations:** 1 DDS, MSc, PhD student. Universidade de Pernambuco, School of Dentistry, Oswaldo Cruz University Hospital, Department of Oral and Maxillofacial Surgery, Recife, Pernambuco, Brazil; 2 DDs, MSc, PhD, Professor. Universidade Federal de Pernambuco, Clinical and Preventive Dentistry Department, Oral Radiology Sector, Recife, Pernambuco, Brazil; 3 DDS, MSc. Universidade de Pernambuco, School of Dentistry, Oswaldo Cruz University Hospital, Department of Oral and Maxillofacial Surgery, Recife, Pernambuco, Brazil; 4 DDS, MSc, PhD. Universidade Federal de Pernambuco, Clinical and Preventive Dentistry Department, Oral Radiology Sector, Recife, Pernambuco, Brazil; 5 DDS, PhD, Professor, Chair of Postgraduate Program in Oral and Maxillofacial Surgery. Universidade de Pernambuco, School of Dentistry, Oswaldo Cruz University Hospital, Department of Oral and Maxillofacial Surgery, Recife, Pernambuco, Brazil

## Abstract

**Background:**

The aim of the present study was to evaluate postoperative effects of platelet-rich fibrin (PRF) in wound and bone healing, pain, swelling and periodontal complications outcomes after impacted third molars extraction.

**Material and Methods:**

A prospective, randomized, split-mouth, double-blind clinical trial was conducted. PRF was placed within sockets following tooth removal and before suturing mucoperiosteal flap while no treatment was performed on control group’s sockets. Patients were evaluated considering bone volume which was obtained in the 90-day postoperative period. Other variables included trabecular thickness, trabecular distance and grey values, pain, swelling, and wound healing. A Wilcoxon test and a t-Student test were used at a 5% significance level and a Friedman test was used to multiple comparisons.

**Results:**

Forty-four surgeries were performed in the present study. The patients’ mean age was 22.41 (± 2.75 years) and 72.73% were women. PRF was associated to increased trabecular thickness and bone volume means (*p* < 0.001). The experimental group had significantly lower pain scores at 4h, 6h, 8h, 16h, 24h, and 72h (*p* ˂ 0.05). Mean swelling was lower on the experimental group (*p* < 0.001). The PRF group showed significant higher wound healing (*p* ˂ 0.001).

**Conclusions:**

Alveolar filling with PRF improves wound and bone healing after extractions while also decreasing pain and swelling in the postoperative period.

** Key words:**Third molar surgery, platelet-rich fibrin, bone regeneration, pain.

## Introduction

Impacted third molars extraction are the most common surgical procedure in the clinical practices of Oral Maxillofacial Surgeons (1-4). The postoperative period after an impacted mandibular third molar (IMTM) surgery is often accompanied by pain, swelling, trismus, periodontal defect distal to the second molars and (5-7), less frequently, alveolar osteitis, nerve damage, and bleeding (2,8,9). Besides a well-planned surgical procedure, other strategies have been adopted to minimize inflammatory response, such as the use of pharmacological methods and blood concentrates (2,5,6).

Due to the release of growth factors, platelet concentrates have been used to improve the healing process and enhance bone repair (2). Platelets contain large amounts of platelet-derived growth factor (PDGF), vascular endothelial growth factor (VEGF), and transforming growth factor (TGF) β1 and β2(10), which stimulate cell proliferation and promote angiogenesis (2,10). The release of growth factors, cell adhesion molecules, and both pro-inflammatory and anti-inflammatory cytokines for up to seven days modulate the inflammatory response. This enhances the efficacy of angiogenesis, neovascularization and tissue regeneration, as well as decreases postoperative pain and swelling (5-7).

The purpose of this study was to evaluate the postoperative effects of platelet-rich fibrin (PRF) in bone healing, wound healing, pain, swelling and periodontal complications outcomes after impacted mandibular third molars extraction.

## Material and Methods

**Figure 1:**
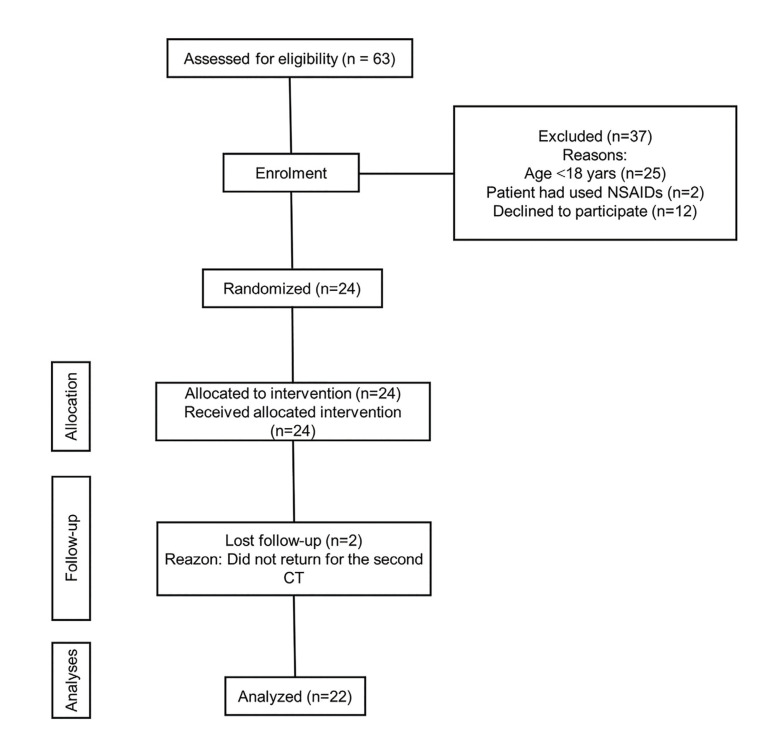
Flow diagram of the randomized clinical trial and the information about volunteer recruitment and follow up.

To address the purpose of this research, the investigators designed and implemented a randomized, split-mouth, double-blind, clinical trial. The study protocol was reviewed and approved by the Local Research Ethics Committee, University of Pernambuco, Brazil (certificate #: 08330919.0.0000.5207) and registered on the (Brazilian registry of clinical trials (ReBEC) number: RBR-64vgjd. All patients gave their written informed consent before randomization. The study was conducted in accordance with the declaration of Helsinki and followed all the protocols of the Consolidated Standards of Reporting Trials Statement (11) (Fig. 1). All the patients received information regarding the study before signing the consent forms.

- Patient Selection

To be included in the study sample, patients had to fulfill the following criteria: 1) indication for impacted third molar extraction; 2) both third molars with same impaction pattern and fully developed roots; 3) adults aged between 18 and 35 years old; 4) the absence of systemic diseases such as hypertension, diabetes mellitus, systemic endocrine disorders, kidney diseases and osteoporosis; 5) the non-use of medication that could interfere with the outcomes of the procedure such as antibiotics, non-steroidal anti-inflammatory drugs or corticosteroids; 6) having no signs or symptoms of infection; 7) having symmetric third molars, and 8) coagulogram showing normal platelet count (150,000-400,000/mm³). Patients were excluded as study subjects if pregnant or breast-feeding, had history of allergy or contraindication to any of the medications used in the study, or were smokers. Besides, patients who did not participate in all phases of the study, and those who did not have the adjacent mandibular second molar were also excluded.

- Study Design

A randomized, split-mouth, double-blind, clinical trial was conducted. On control group socket was sutured with the clot alone and in PRF group clots from two tubes were applied to the socket prior to the final suture. All the surgical procedures were performed by the same surgeon (E. D. R. R.).

Randomization regarding the experimental and control sides was performed using a computer-generated randomization code (Microsoft Office Excel® 2010). The randomization data were only known to the surgeon. Patients, the clinician that performed all clinical evaluations (R. A. P. M), the radiologist (A. A. P) and the statistical expert (B. C. E. V.) were blinded to the randomization data until the final analysis of the experiment.

Initially, ten patients underwent mandibular third molar extractions (20 extractions) in order to determine the sample size. The sample size was estimated considering the standard deviation of the mean differences in bone volume between the experimental and control groups (4.402 mm³) and the standard deviation [4.364]. The sample size was calculated considering an alpha level of 0.05, a test power of 80% and a 95% confidence interval. Based on this data, the estimated optimum sample was 16 patients.

- Surgical Technique

The surgical procedures were performed under local anesthesia (buccal and lingual infiltration and inferior alveolar nerve block). An incision was made in the distal region of the gingival sulcus of the second molar; an oblique mesial side incision of this tooth was then made to create the mucoperiosteal flap. Osteotomy and tooth sectioning were done with rotary instruments under manual irrigation. The third molar was extracted, and the surgical wound was cleaned by irrigation with 0.9 % saline. All patients received instructions regarding their diet and oral hygiene.

In then PRF group, clots from two tubes were applied to the socket prior to the final suture, whereas in the control group, the socket was sutured with with the clot alone. The flap was repositioned and sutured with 4-0 silk thread (Ethicon). The suture was removed one week after surgery.

Postoperatively, we prescribed a 0.12% chlorhexidine digluconate mouthwash for 7 days (15ml/1min/q12hr). Paracetamol (acetaminophen) 750 mg was provided to participants experiencing pain (≥ 3 score) within 4 hours after surgery as a rescue drug and every 6h thereafter.

- Clinical Assessment and Outcomes

The predictor variable was the treatment (experimental vs. placebo). Patients had both lower third molars extracted at the same surgical time, and each side was randomly allocated to: 1) the experimental group, in which the socket was filled with a PRF plug; and 2) the control group, in which no filling was received (the opposite side).

Bone volume was the primary outcome variable. The secondary outcome variables were trabecular thickness, trabecular distance, greyscale, pain, swelling and wound healing. and probing pocket depth.

**Figure 2:**
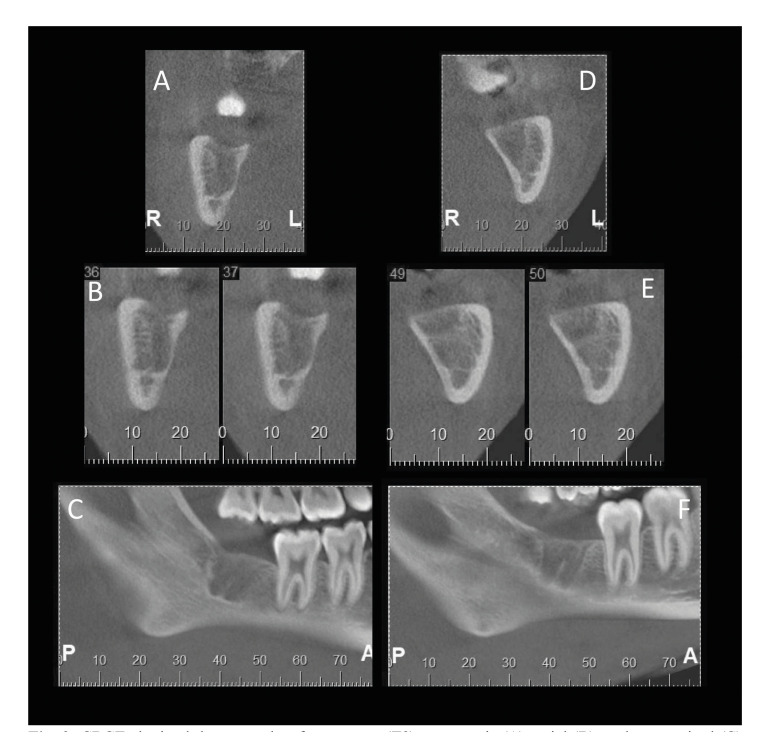
CBCT obtained three months after surgery (T3). panoramic (A), axial (B), and parasagittal (C) reconstructions showing the bone repair of a L-PRF socket and panoramic (D), axial (E), and parasagittal (F) reconstructions showing the bone repair of control a socket (same patient).

Cone beam computed tomography (CBCT) was performed using the i-CAT Next Generation (Imaging Sciences International, Pennsylvania, USA) operating at 120 kVp, 18.5 mAs, field of view (FOV) of 6 x 16 cm and 0.25 mm voxel size. CBCT images of each patient were obtained three months after surgery (T3) and exported for evaluation in ImageJ/Fiji software (National Institutes of Health, Bethesda, MD, USA). All CBCT evaluations were performed by an oral and maxillofacial radiologist with 10 years of experience. The images were registered with the Registration/Rigid 3D tool. A cubic volume of interest (VOI) (4mm in height, 4mm in length, and 4mm in depth) was determined over the medullary bone at the base and center of the socket. The standardization of this VOI was achieved with the ROI Manager function of ImageJ/Fiji software. All images were then converted into eight bits and binarized using the "Moments” automatic binarization method (12-14). The BoneJ plugin was used to evaluate bone volume, trabecular thickness and trabecular distance (Fig. 2). The mean and standard deviation values of the greyscale within each VOI were determined by histogram analysis.

**Figure 3:**
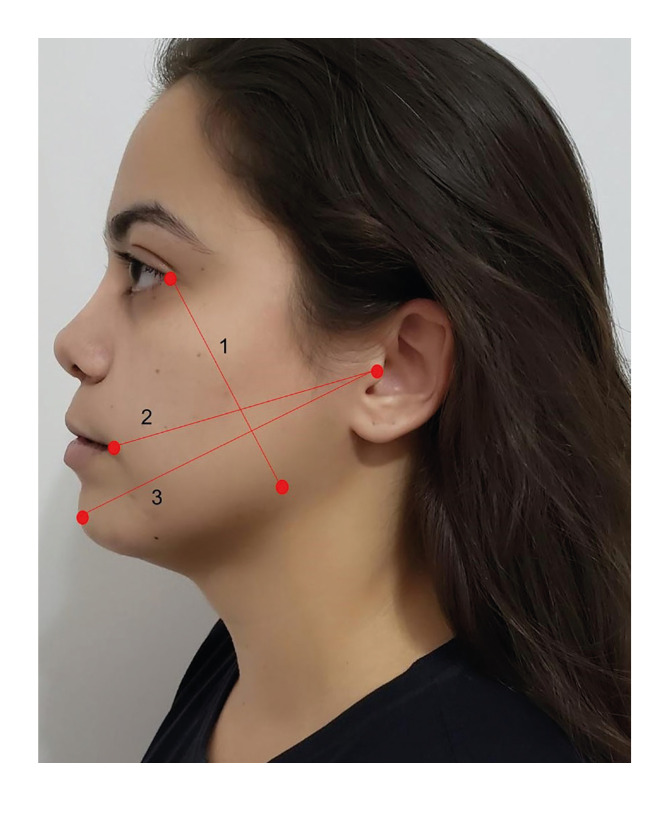
Swelling measurements.

The postoperative pain assessment was performed by the visual analog scale (VAS) using a 100 mm scale ranging from 0 (no pain) to 10 (maximum imaginable pain). The patients were asked to indicate the intensity at 30 minutes and at 2, 4, 6, 8, 12, 16, 24, 48 and 72 hours after the surgery, in the postoperative period. The swelling was assessed by comparing the values of three facial lines: 1) the distance from the lateral corner of the eye to the gonial mandibular angle; 2) the distance from the lowest point of the tragus to the mouth commissure; and 3) the distance between the lowest point of the tragus to the pogonion (Fig. 3). These measurements were taken before surgery (T0) as well as 72 hours (T1) and seven days (T2) after the surgery by a collaborating clinical evaluator that also was blinded to the randomization of the experimental and control groups. We used the sum of the three measurements to quantify swelling. At T1 and T2, a clinical evaluation was performed to determine postoperative wound healing using the modified Landry index (15). A millimeter periodontal probe (Carolina do Norte, Hu-Friedy®, Chicago, IL, USA) was used to measure probing pocket depth on three sites around the second molar (distobuccal, distolingual and distal), as described by Kumar *et al* (16). All clinical assessments were made by a single clinician. Demographic variables (sex and age), third molar surgery operative times and impaction pattern of the teeth Pell and Gregory's classification were also recorded.

- PRF protocol

Blood samples were taken in 10-mL tubes without anticoagulant. The tubes were centrifuged at 2700 rpm (approximately 400 g) for 12 minutes in a Daiki DT4000 centrifuge according to the method described by Dohan *et al*.(17).

- Data analysis

Data were expressed as the mean, standard deviation, minimum and maximum values. To assess data normality, we conducted a Shapiro-Wilks test. For the normally distributed postoperative times variables, a t-Student test for paired samples was used to compare PRF and control groups at different postoperative times. In contrast, for the data that were not normally distributed, the Wilcoxon test was performed. The data were statistically analyzed using SPSS v. 20.0 (IBM Corp., Armonk, NY) with a significance level of 5%.

## Results

A total of 61 patients were recruited for the study. Thirty-seven did not meet the eligibility criteria and 24 were selected for the intervention. Two patients did not return for the postoperative evaluations and were excluded from the study. Thus, the final sample was composed of 22 patients, as shown in the flow diagram (Fig. 1). Patients’ age ranged between 18 to 28 years (22.41 ± 2.74 years). Seven (31.8%) were male and 15 were female (68.2%). A total of 44 third molars were classified according to Pell and Gregory's (18) and Winter's (19) classifications as 1B = 16 (36.4%), 1C = 12 (27.3%), 2A = 8 (18.2%), 2B = 2 (4.5%) and horizontal = 6 (13.6%). The right sides were randomly allocated to the control group in 13 individuals while the left side was thus allocated in 9 individuals.

There were no statistically significant differences among demographic variables. Mean operative times of third molar surgery were 21.6 minutes (± 2.1) in the control group and 21.4 minutes (± 1.6) in the experimental group and it did not differ in more than 5 minutes between each side of the same patient. Regarding these operative times, there was no statistically significant differences between the different sides of the same patient.

The PRF group showed significantly higher wound healing scores at the third day (2.23 - poor) when compared with the control group (1.73 - very poor) (*p* = 0.001) (Table 1). On the seventh day, the experimental group also had a significantly higher mean score (4.23 - very good) compared to the control group (3.18 - good) (*p* = 0.001).

The patients began to report pain four hours after surgery (Table 2). Regarding postoperative pain, peak scores occurred on the second day followed by a progressive reduction through to the seventh day. Statistically significant pain scores differences between PRF and control groups were found at 6 h (*p* = 0.001), 8 h (*p* = 0.001), 16 h (*p* = 0.025), 24 h (*p* ˂ 0.001), 48 h (*p* ˂ 0.001) and 72h (*p* ˂ 0.001), with the control side showing higher pain scores.

Regardless of the group, swelling peak occurred 72h after surgery (Table 3). The PRF sides exhibited statistically significant lower means at this postoperative time (*p* ˂ 0.001). No periodontal defects were found distally to the mandibular second molar on the seventh day or on the 90th day after the impacted third molars extractions (probing depth ≤ 3 mm).

Tomographic evaluation showed significantly higher trabecular thickness and bone volume in the experimental group (*p* ˂ 0.05) (Table 4). Concerning trabecular distance and grey values means, we found no statically significant differences between experimental and control groups (*p* > 0.05).

**Table 1:**
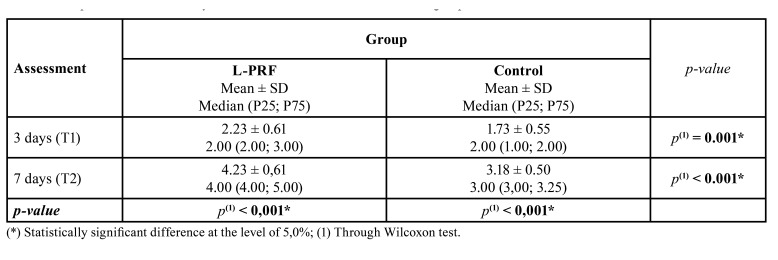
Comparison of mean Landry index values between L-PRF and control groups.

**Table 2:**
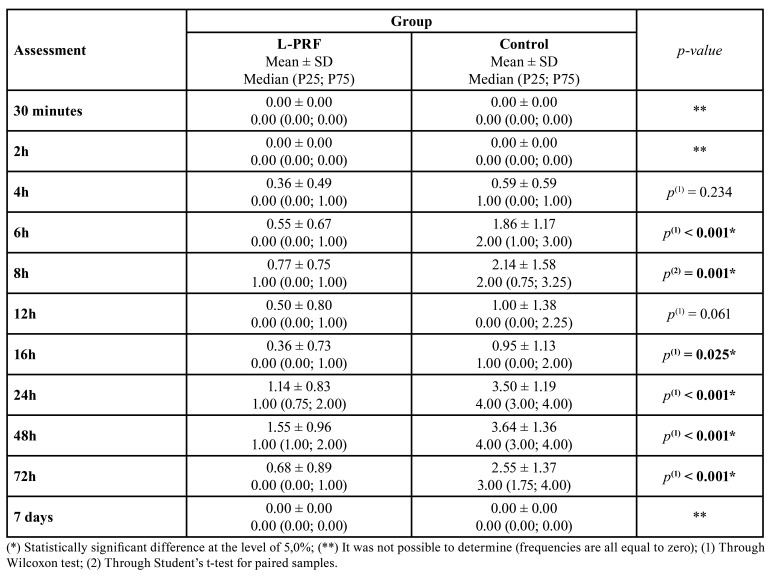
Comparison of pain scores (VAS) between L-PRF and control groups at different postoperative times.

**Table 3:**
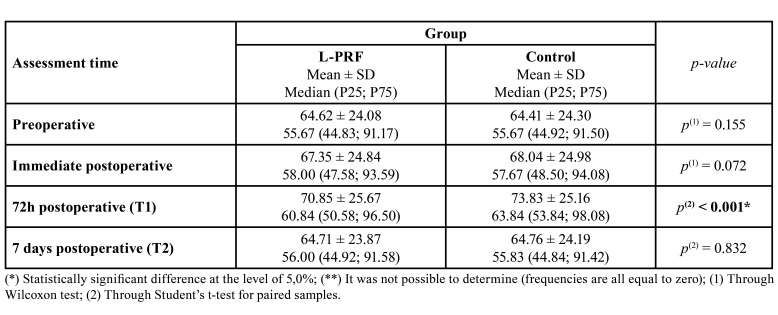
Comparison of mean swelling values between L-PRF and control groups.

**Table 4:**
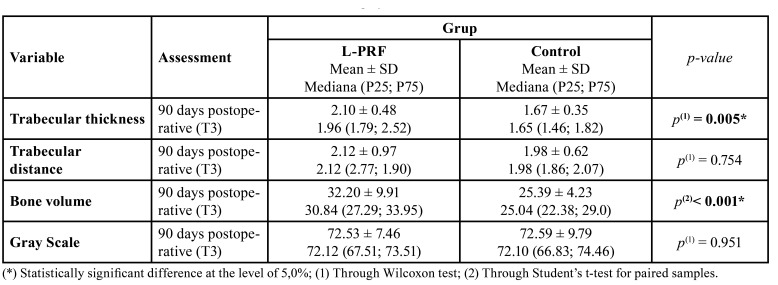
Measurements of trabecular thickness, bone volume and gray scale.

## Discussion

The purpose of this study was to evaluate the postoperative effects of PRF in bone healing, wound healing, pain, swelling and periodontal complications outcomes after impacted mandibular third molars extraction. We hypothesized that PRF would enhance bone repair and would modulate the inflammatory response. The specific aims of the present study were 1) to measure differences in bone volume, trabecular thickness and greyscale for the two different treatment groups and 2) to evaluate the efficiency of PRF in terms of swelling reduction, pain and wound healing improvement. The results indicated that the use of PRF in impacted third molar sockets after the extraction significantly improves bone healing on the 90-day postoperative evaluation.

Third molar extraction is the most common procedure performed on the clinical routine of Oral Maxillofacial Surgeons (5-7), which was confirmed by the treatment demand observed in the present study. To reduce interpersonal variation, our study used the split mouth design since bilateral extraction of impacted mandibular third molar using a split-mouth design is a commonly used experimental model, as it eliminates this variation (9).

The healing process, swelling, and pain can be affected by several factors, such as surgeon experience, degree of trauma, patient age and gender, and third molar surgery operative time (16,18,19). In our study, all surgeries were performed by the same experienced surgeon and there were no significant differences between the operation times of either side. Additionally, our study included only third molars with same impaction pattern on both sides, and fully developed roots in order to standardize surgical procedures.

In the analysis of the modified Landry Index (15) for determining soft tissue healing, the results favored the use of PRF. These findings are in agreement with data reported in a systematic review performed by Miron *et al*.(20) in which the majority of studies demonstrated favorable PRF results with regards to soft tissue healing. Yelamali *et al*.(19) and Singh *et al*.(21) also reported similar results. Leukocytes play a fundamental role in the healing of injuries due to the anti-infection action and immunological regulation through the secretion of immune cytokines, such as interleukin (IL)-1β, IL-4 and IL-6, and tumor necrosis factor alpha (TNF-α) (22,23). Fibrin forms a temporary matrix that enables cellular invasion and tissue regeneration (24). Platelets play a fundamental role in hemostasis and are a natural source of growth factors, such as PDGF, VEGF, TGF-β1 and TGF-β2 (10,19), which stimulate cellular proliferation and angiogenesis (2,10). This may explain the favorable soft tissue healing results in the present study.

The application of platelet concentrates following third molar extraction has been described in the literature with promising results (2,5,6,8,16,18,19). Compared to other concentrates, PRF has advantages, as it is easily prepared and does not require the use of anticoagulant (6). Besides, PRF contains 97% of platelets and more than 50% of leukocytes within a highly dense fibrin network when compared to total blood (10). The use of L-PFR as a biomaterial in oral surgical procedures requires an initial investment to acquire a specific centrifuge. However, PRF can be produced by any modified laboratory centrifuge (10,14). Once the centrifuge is purchased, the production of PRF does not represent a significant cost as blood collection equipment are quite inexpensive (less than $ 10).

In some clinical trials (8,16,18), postoperative pain peaks occurred between 24 and 48 hours after the procedure, which is similar to our findings. This significantly lower pain found on the experimental side is ratified by the findings of Kapse *et al*.(8) and Kumar *et al*.(16) Besides, in a systematic review performed by Al-Hamed *et al*.(7), two of the three studies reported a significant reduction in postoperative pain.

Regarding postoperative swelling and pain, the PRF group showed earlier pain and swelling reduction during peak (72 h). This finding agrees with data reported in previous studies (6,16). He *et al*.(2) found no significant difference in swelling between control and PRF groups on the first nor on the second day after third molar extractions, but they also found a significant difference at 72 h. Therefore, we may state that the use of PRF reduces swelling at 72 hours after impacted mandibular third molar extraction. The α-granules of platelets progressively release the cytokines and growth factors in the PRF clot on implanted sites during the fibrin matrix’s remodeling phase. That may explain pain and swelling reduction in the PRF group in the present study.

In our study, no periodontal defects adjacent to the second molar were observed in either group. Ritto *et al*. (15) also found no statistically significant differences in probing depth between the control and experimental groups, which is in agreement with the present findings. However, previous studies have shown that impacted third molar extraction can result in periodontal defects distally to the mandibular second molar and that PRF is effective in inducing and accelerating bone repair (9). Kumar *et al*.(16) evaluated probing depth in the distal region of the second molar one and three months after extraction and found that the PRF group had shallower probing depths, but the difference was only significant at the three-month evaluation.

A previous study (15) measured the grey values in order to evaluate bone healing and found that the PRF group had higher grey value mean and stated that higher grey values are an indication of higher bone density. In the present study, CBCT was chosen in order to enable a structural bone analysis in addition to measuring grey values, since the inaccuracies in grey values may lead to inconsistencies on bone density measurements. Also, some limitations have been linked to the use of grey values for bone quality assessment in clinical use (13). For instance, grey values measurements of bone may be influenced by the presence of other adjacent anatomical structures such as the vertebral column (25) and vary according to anatomical location (26). Therefore, it is not certain that the grey values derived from CBCT represent the real bone density values, which differs from Multidetector Computed Tomography density values (CT number densities termed Hounsfield units).

The use of CBCT images enables the analysis of bone structure parameters usually performed in micro-CT or histology (13). In contrast, prior clinical trials (8,16) performed this evaluation using two dimensional radiographs, but they didn’t point out the inherent bias found in this image modality. Failure to do so can lead to erroneous interpretations, as two-dimensional radiographs are significantly less accurate in the evaluation of small defects in comparison to cross-sectional reconstructions (27). The present findings reveal significant improvements in trabecular thickness and bone volume in the PRF group. Baslari *et al*. (28) using scintigraphy to assess bone repair, demonstrated no increase in osteoblastic activity in PRF filled alveolae. A possible explanation for our findings is the secondary role played by cytokines and growth factors, which don’t seem to enhance the proliferation of bone tissue cells, but rather favor regional vascularization through angiogenesis (29). A systematic review revealed that the use of PRF promoted better bone repair, but a meta-analysis was not possible due to the considerable methodological heterogeneity among the studies (30). Similar findings are described in a systematic review performed by Miron *et al*. (20) who concluded that PRF improves tissue regeneration and may limit dimensional changes following tooth extraction. Our findings suggest that PRF improves bone repair after extractions, which may support this approach in future rehabilitation treatments with dental implants and bone graft procedures after tooth loss, since it is a low-cost autologous biomaterial with an easy and simple preparation.

When discussing the limitations of our study, we cannot exclude the possible effects of rescue medication intake on postoperative pain levels. In this study, the patients received oral and written instructions regarding the use of acetaminophen. Pain subjectivity is a complicating factor in the patient’s understanding in regards to the need to take a rescue medication in the postoperative period. Postoperative wound healing evaluation was performed using a modified Landry index (15). The subjectivity of this analysis is a limitation. Just young and healthy individuals with both mandibular third molars in the same position were included in this study. Furthermore, the same surgeon performed all the surgeries. Both appraiser (clinician and radiologist) and patients were blinded, in an attempt to improve results’ accuracy. Surgeries on both teeth were performed during the same operative time, so we cannot evaluate the PRF effects on trismus.

The use of PRF in impacted third molar sockets after the extraction significantly improves bone healing as it exhibits higher trabecular thickness and bone volume on the 90-day postoperative evaluation. PRF reduces postoperative pain and swelling and improves wound healing during the postoperative period. Besides, it does not affect the distal probing depth of the second molar on the 90th day of postoperative period.
